# Microarray Expression Analysis of the Main Inflorescence in *Brassica napus*

**DOI:** 10.1371/journal.pone.0102024

**Published:** 2014-07-09

**Authors:** Yi Huang, Jiaqin Shi, Zhangsheng Tao, Lida Zhang, Qiong Liu, Xinfa Wang, Qing Yang, Guihua Liu, Hanzhong Wang

**Affiliations:** 1 Oil Crops Research Institute, Chinese Academy of Agricultural Sciences, Wuhan, Hubei, P. R. China; 2 Plant Biotechnology Research Center, School of Agriculture and Biology, Shanghai Jiao Tong University, Shanghai, P. R. China; 3 School of Life Science and Technology, Hubei University, Wuhan, P. R. China; New Mexico State University, United States of America

## Abstract

The effect of the number of pods on the main inflorescence (NPMI) on seed yield in *Brassica napus* plants grown at high density is a topic of great economic and scientific interest. Here, we sought to identify patterns of gene expression that determine the NPMI during inflorescence differentiation. We monitored gene expression profiles in the main inflorescence of two *B. napus* F_6_ RIL pools, each composed of nine lines with a low or high NPMI, and their parental lines, Zhongshuang 11 (ZS11) and 73290, using a *Brassica* 90K elements oligonucleotide array. We identified 4,805 genes that were differentially expressed (≥1.5 fold-change) between the low- and high-NPMI samples. Of these, 82.8% had been annotated and 17.2% shared no significant homology with any known genes. About 31 enriched GO clusters were identified amongst the differentially expressed genes (DEGs), including those involved in hormone responses, development regulation, carbohydrate metabolism, signal transduction, and transcription regulation. Furthermore, 92.8% of the DEGs mapped to chromosomes that originated from *B. rapa* and *B. oleracea*, and 1.6% of the DEGs co-localized with two QTL intervals (*PMI10* and *PMI11*) known to be associated with the NPMI. Overexpression of *BnTPI*, which co-localized with *PMI10*, in *Arabidopsis* suggested that this gene increases the NPMI. This study provides insight into the molecular factors underlying inflorescence architecture, NPMI determination and, consequently, seed yield in *B. napus*.

## Introduction

The inflorescence architecture of plants is a key agronomic factor determining seed yield, and is thus a major target of crop domestication and improvement [1], [2]. Variations in agricultural productivity and reproductive success are largely determined by differences in shoot architecture, and reproductive shoots known as inflorescences exhibit tremendous diversity across flowering plants in both branch and flower number [3]–[5]. The inflorescence architecture depends on developmental decisions at the inflorescence meristem [6], [7].

*Brassica napus* is one of the most important cash crops in the *Brassicaceae* family. The transition from vegetative to reproductive growth is particularly important for successful seed production. After the floral transition, the main inflorescence (MI) meristem either acquires a floral meristem identity or produces lateral meristems, which further iterate the MI meristem pattern (Benlloch et al., 2007; Bradley et al., 1997; Prusinkiewicz et al., 2007; Thompson and Hake, 2009; Wang and Li, 2008). Since the number of pods on the main inflorescence (NPMI) influences the productivity of *B. napus*, especially under high-density growth conditions, plant breeders throughout the world are interested in identifying the factors that determine the initiation and formation of pods on the MI. Therefore, understanding the genetic basis of inflorescence architecture will not only elucidate this intriguing evolutionary mechanism, but may also be used to improve crop grain yield [1], . Although the link between the NPMI and yield is well established, surprisingly little research has examined the molecular basis of the differentiation and development of pods on the MI in *B. napus*. Such information is essential for improving molecular breeding techniques and developing genetic modification strategies.

Several lines of genetic evidence suggest that inflorescence architecture is controlled by multiple genes and their interactions [9]–[12]. Transcriptome analysis provides a valuable tool for identifying the network of genes underlying MI development. Microarray analysis and transcriptomic sequencing have been used to identify genes involved in numerous biological processes in several species [13]–[19]. Transcriptomic sequencing is widely used for genome-wide expression pattern analysis in species that have been fully sequenced, such as *Arabidopsis* and rice. Conversely, microarray analysis is suitable for identifying genes in species such as *B. napus*, for which little reference genome information is available. Recently, an oligonucleotide array of *Brassica* harboring 90K genes was developed in CombiMatrix and used to identify genes in *B. napus* siliques subjected to drought stress [20] and to decipher the biology of seed coats in canola [21].

To gain insight into the mechanism underlying inflorescence shaping, the differentiation and development of pods on the MI, and high yield formation in *B. napus*, we adopted a microarray-based approach to study large-scale gene expression changes in the inflorescence. Specifically, we investigated the transcriptomic variation at the pistil-stamen primordial differentiation stage of two *B. napus* F_6_ RIL pools, each composed of nine lines with an extremely low or high NPMI, and their elite parental lines, Zhongshuang 11 (ZS11) and 73290, which exhibit marked differences in the NPMI, using the 90K oligonucleotide array. We sought to identify patterns of gene expression in the MI during pod differentiation, determine biological processes associated with pod differentiation and determination, and identify potential candidate genes that are involved in inflorescence development, and especially in pod determinacy, by identifying genes that are both differentially expressed in the MI and co-localized with QTL intervals associated with the NPMI. Furthermore, we planned to demonstrate the functional roles of some representative potential DEGs in increasing the NPMI by overexpressing these DEGs in *Arabidopsis* wild type plants.

## Materials and Methods

### Plant growth and sample collection

Two elite *Brassica napus* L. lines, cv. Zhongshuang 11 (ZS11), which was used for genome sequencing, and 73290, which was used for re-sequencing, developed by the Rapeseed Biotechnology Breeding Unit, Oil Crops Research Institute of the Chinese Academy of Agriculture Sciences (Wuhan, China), were used to generate an F_2∶3_ population harboring 183 offspring for QTL mapping of yield traits, as previously described [22]. Subsequently, an F_6_ inbred line (RIL) population was obtained from the F_2∶3_ population by single seed descent. Twelve lines with an extremely high number of pods on the main inflorescence (NPMI) and 11 with an extremely low NPMI were selected from the above F_6_ RIL population. Four of these lines flowered too late to harvest and one was partially sterile, leaving nine high- and nine low-NPMI lines. The ZS11, 73290, and 18 RIL lines were used for gene expression profiling analysis in the main inflorescence (MI).

Seeds were sown on May 17, 2011 in the experimental station of Qinghai University (Xining, Qinghai Province, China), which is managed by local people. The owner provided full access permission to the experimental site. The field trails did not range over any protected or endangered species, and no vertebrates were involved in this study. Individual plants in each line that did not exhibit any obvious phenotypic differences were selected at several developmental stages, to avoid the erroneous inclusion of plants not belonging to the line. The development of the MI was observed under a microscope (Olympus, Japan). The inflorescence primordia of three to five plants for each line were examined every three days under an anatomical microscope, beginning at the eighth leaf stage. The MI primordia of five plants per line in which the first flower on the MI was in the pistil-stamen primordia initiation stage were harvested in the morning, immediately frozen in nitrogen, and then kept at −80°C for total RNA isolation. Ten mature plants of each line were harvested and the inflorescence traits were recorded.

Seeds of *Arabidopsis thaliana* ecotype Col-0, transgenic *D35S::BnTPI* and *pBnTPI::GUS*, and empty vector (EV) lines were germinated on MS (Murashige & Skoog) medium in Petri dishes for 4 days at 4°C in the dark, and then transferred to a growth room at 21°C under a regime of 16-h light/8-h dark at a light intensity of ∼150 µE^.^m^−2.^s^−1^. After 7 to 10 days, the seedlings were transplanted to pots of soil (Peilei Co., Jiangsu, China) and grown under the same conditions as described above. Transgenic and control lines were grown under identical conditions in different pots to avoid cross-pollination.

### RNA extraction

Using an RNeasy Mini Kit (Cat. 74124, Qiagen, Mississauga, ON) according to the manufacturer's recommendations, the total RNA of inflorescence primordia was extracted in biological triplicate, each consisting of three main inflorescence sections taken from three independent plants. Trace amounts of DNA were removed using DNase I (Cat. 18068-015, Invitrogen, USA). The RNA yield and purity were determined spectrophotometrically with a NanoDrop 1000 spectrophotometer (Thermo Fisher Scientific, USA), and the intactness of RNA was verified by electrophoresis on a 1% agarose gel with 1×TBE running buffer at 70 V for 40 min. Purified total RNA was precipitated and re-suspended in DEPC-treated water to a final concentration of about 500 ng/µL. For the high- and low-NPMI lines, the total RNA of inflorescence primordia was extracted individually and pooled at equal concentrations within each extreme-NPMI group. Nine lines were used for each biological replicate in the reverse transcription analysis.

Total RNA of *Arabidopsis* transgenic and control seedlings was extracted using an RNAprep Pure Plant Kit (TIANGEN, China) under the manufacturer's recommendations.

### Microarray analysis

#### 1) *Brassica* 90K array

A *Brassica* genomics resource was developed at the Plant Biotechnology Institute, National Research Council Canada (PBI-NRC) and Agriculture and Agri-Food Canada (AAFC) in collaboration with other institutes. About 95,418 unique sequences were assembled using 781,826 EST sequences mainly from three species: *B. napus*, *B. rapa*, and *B. oleracea.* These unique sequences were submitted to CombiMatrix for the development of a 35–40mer oligonucleotide microarray. Probes were synthesized *in situ* on electrodes of a microchip. The customized CombiMatrix *Brassica* array comprised 90,500 probes and was named the Brassica 90K array.

#### 2) aRNA in vitro synthesis, dye coupling, and hybridization to the *Brassica* 90K array

cDNAs were synthesized from total RNA extracted from the main inflorescence of ZS11 and 73290, and from the high- and low-NPMI pools. About 3.0 µg of total RNA for each sample was reverse transcribed into amino allyl-modified aRNA using a Message II aRNA Amplification Kit (Cat: 1753, Ambion, Austin, TX, USA), according to the manufacturer's protocol. The amplification products were analyzed by agarose gel electrophoresis and ethidium bromide staining, and the aRNA yield was quantified using a NanoDrop 1000 spectrophotometer. The aRNA was selected as template for fluorescent target preparation for microarray experiments.

About 5.0 µg of aRNA was labeled with mono-reactive NHS esters of Cy5 in the dark for 30 min at room temperature, and the samples were purified and used as targets for hybridization. The pre-hybridization and hybridization procedures were performed precisely according to the protocol of CombiMatrix (www.combimatrix.com). Cy5 dye-labeled RNA populations from individual tissue samples were hybridized to the 90K array and three biological replicates were performed.

#### 3) Data collection and analysis

Hybridized arrays were scanned using a LuxScan 10K Scanner (CapitalBio Corporation, Beijing, China) at 5 µm resolution, 100% laser power, and different PMT values to obtain a similar overall intensity between slides. The scans were saved as TIF files. Raw spot fluorescence intensities were collected using LuxScan version 2.0 (CapitalBio Corporation, Beijing, China) and saved as LSR files. The microarray dataset generated in this study was deposited in NCBI's Gene Expression Omnibus and is accessible through GEO series accession number GSE57886 (http://www.ncbi.nlm.nih.gov/geo/query/acc.cgi?acc=GSE57886).

Before normalization, basic pre-processing was performed. Samples that were originally negative after background subtraction and those with an overall signal intensity of ≤100 were filtered out. Array features annotated as “Empty”, “Blank” and those internal controls were flagged and excluded from the analysis. Twelve LSR files containing the raw probe intensity values were imported into R and the signal intensities were subjected to quantile normalization to normalize between arrays using the R-package LIMMA [23] with a 5% false discovery rate (FDR≤0.05). Probes with at least one missing value within triplicates were removed from subsequent analysis. The background-corrected signal intensity of each probe on the array was combined by averaging three biological replicates. A gene was considered to be differentially expressed when its fold-change in expression between two samples was ≥1.5 or ≤0.67.

Probe annotation analysis was performed using BLASTx at an *E*-value of ≤10^−5^ or a BLASTn score of ≥100 against the TAIR10 database (http://www.arabidopsis.org). The functional categories were calculated using bootstrap analysis with 100 replicates of the TAIR10 Arabidopsis genome annotation, at a 95% confidence level (p<0.05), and MapMan was used as the classification source [24].

### Isolation and cloning of *BnTPI*

#### 1) Construct preparation and transformation

The entire open reading frame (ORF) of *BnTPI* was amplified by RT-PCR using a pair of gtpi primers from the cDNA of ZS11, resulting in the *pD1301S::BnTPI* construct with a 765-bp insert. The *pBnTPI::GUS* construct harbored a 1,706-bp fragment of the *BnTPI* ORF that had been amplified with ptpi primers from the cDNA of ZS11. PCR was carried out with a C1000 PCR system (BIO-RAD) using an *Easy Taq* DNA Polymerase Kit (Cat: E51117, TransGen, China) and the following amplification cycle: 30 cycles of 94°C for 30 s, 58°C for 30 s, and 72°C for 40 s. The amplicons were collected and cloned using TA-overhangs into the pMD18-T vector (Takara). The integrity of cloned inserts was confirmed by sequencing with a pair of M13 primers. These coding regions of *BnTPI* in pMD18-T were respectively transferred to generate expression plasmids *pD1301S::BnTPI* and *pBnTPI::GUS*, which were confirmed by PCR using a pair of primers based on the cauliflower mosaic virus 35S constitutive promoter (35s-f) and gtpi-r, and the pC1301GT (cgt-r) and ptpi-f primers, respectively. The sequences of all the PCR primers are listed in Table S3.

*Agrobacterium tumefaciens* strain GV3101 harboring *pD1301S::BnTPI* or *pBnTPI::GUS* was transformed into *Arabidopsis thaliana* wild-type Col-0 plants by floral dip as previously described [25]. Transformants were germinated and screened based on hygromycin B resistance on Murashige and Skoog medium.

#### 2) Real-time PCR

Total RNA of transgenic and control seedlings was extracted using the RNAprep Pure Plant Kit (TIANGEN, China) under the manufacturer's recommendations. First-strand cDNA was synthesized from 10.0 µL of total RNA using Oligo (dT) and 200 units of M-MLV reverse transcriptase (Promega) and incubated at 70°C for 5 min, in an ice-bath for 5 min, and at 42°C for 60 min. For comparative PCR, different cDNAs were normalized using *ACTIN2* (AT3G18780)-specific primers (Table S3). Reactions were performed in a final volume of 20.0 µL containing 10.0 µL of 2×SYBR Green Master Mix (TOYOBO), 0.5 mM of each primer, and 2.0 µL of cDNA, using a real-time PCR system (CFX96, Bio-RAD). PCR conditions were as follows: 95°C for 2 min, followed by 40 cycles of 95°C for 15 s, 58°C for 15 s, 72°C for 25 s, and 25°C for 30 s. *ACTIN2* expression was used to normalize the transcript level in each sample. Data were analyzed using sequence detector software (Bio-RAD, Version 2.1).

#### 3) GUS Staining

Nine-day-old, light-grown seedlings expressing the *pBnTPI::GUS* fusion were vacuum infiltrated for 5 min and then incubated overnight at 37°C in reaction buffer containing 50.0 mM sodium phosphate (pH 7.0), 0.5 mM ferricyanide, 0.5 mM ferrocyanide, 0.05% Triton X-100, and 1.0 mM X-Gluc [26]. Plantlets were depigmented with 70% ethanol and GUS staining patterns were documented using a digital camera [27].

## Results

### Morphological differences in the inflorescences of *B. napus* lines

**Figure 1:**
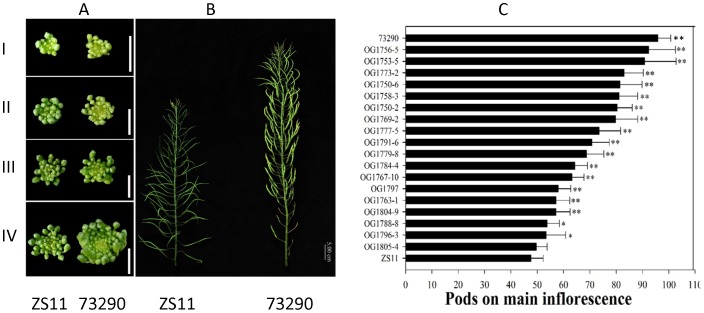
Morphological features of the main inflorescence (MI). (A) Top view of young floral buds on the MI of ZS11 and 73290 at four development stages, namely sepal initiation (I), pistil-stamen initiation (II), petal initiation (III), and pistil-stamen elongation (IV). (B) The intact MI of ZS11 and 73290 before harvesting. (C) Histogram indicating differences in the NPMI between ZS11, 73290, and the F_6_ RIL lines. Significance was determined using Student's *t-*test (n = 30). Asterisks above the columns indicate significant differences compared to ZS11. *, p<0.05; **, p<0.01.

The MIs of plants of line 73290 were much larger than those of ZS11 (Figures 1A and 1B). Furthermore, line 73290 had more differentiated flower buds and a higher NPMI than ZS11 and a longer MI. The NPMI is determined by the duration and rate of differentiation. We found that line 73290 had a longer period of differentiation than did ZS11. A Student's *t*-test (p<0.05) showed that the difference in the NPMI between ZS11 (80∼90) and 73290 (140∼150) was significant. Consequently, we constructed genetic linkage maps using SSR markers to detect QTLs associated with multiple yield-related traits from 183 lines within the F_2∶3_ population derived from the cross between ZS11 and 73290 (unpublished).

Based on the variation in the NPMI in lines within the F_6_ RIL populations, we selected a subset of 23 F_6_ RIL lines that had an extremely low or high NPMI. We counted the NPMI in the ZS11, 73290, and F_6_ lines using 10 mature plants per accession. Differences between ZS11 and all accessions except line OG1805-4 were significant (Student's *t*-test; p<0.05) (Figure 1C), as were those between 73290 and all other accessions examined.

We selected 9 RILs with a high NPMI and 9 with a low NPMI to construct high- and low-NPMI RNA pools for gene expression profiling of the MI using the Brassica 90K oligonucleotide array. Hereafter, we refer to ZS11 and the low-NPMI RNA pool as the low-NPMI sample, and 73290 and the high-PMI RNA pool as the high-NPMI sample.

### Gene expression profiles of the main inflorescence

**Figure 2:**
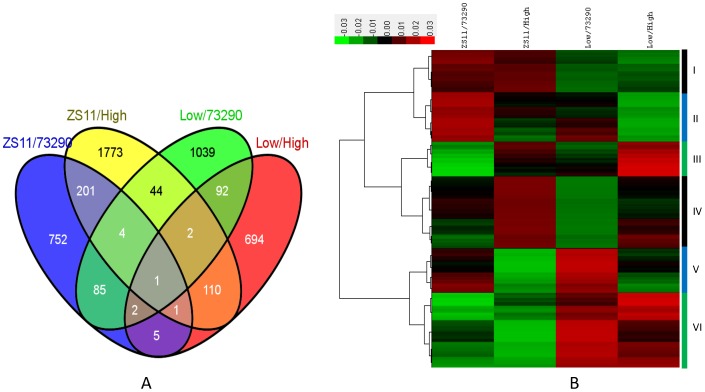
Transcriptomic analysis of the MI of ZS11, 73290, and the two F_6_ pools. (A) Venn diagram of differentially expressed genes (DEGs) between the low-NPMI and high-NPMI samples (p<0.05, absolute fold change of ≥1.5). (B) Hierarchical cluster analysis of genes that were detected as being differentially expressed in at least one of the low-NPMI versus high-NPMI pairs. Red indicates up-regulation and green denotes down-regulation. I–VI indicate the six clusters, and the color bars on the right denote the range of each cluster. Low and High indicate the Low-NPMI and High-NPMI RNA samples, respectively.

As previously described [21], each oligonucleotide probe on the array was designed based on non-overlapping *Brassica* EST contigs and singletons that ideally represent unique genes. For ease of description, we will refer to each of the 90K probes on the array as a gene, and provide AGI locus numbers of the *Arabidopsis* sequences with the highest level of identity for each cDNA or contig that was used as a source for each array probe. A total of 84970 genes produced hybridization signals in all four RNA samples within the biological triplicates and these signals were subjected to data processing. We sought to identify differences in gene expression that led to differences in the MI and NPMI between the low-NPMI and high-NPMI samples, by comparing the expression of genes in the ZS11 vs. 73290 or high-NPMI RNA pool and the low-NPMI RNA pool vs. 73290 or the high-NPMI RNA pool. We identified 1051 and 2136 genes with 207 overlaps that were differentially expressed between ZS11 and 73290, as well as between ZS11 and the high-PMI RNA pool. Similarly, 907 and 1269 genes, including 97 common genes, showed expression differences between the low-NPMI and high-NPMI RNA pool or between the low-NPMI RNA pool and 73290, respectively. Furthermore, 1051 and 1269 genes, with 92 overlaps, displayed expression differences between ZS11 vs. 73290 and between the low-NPMI RNA pool vs. 73290, respectively, and 2136 and 907 genes, with 114 overlaps, exhibited expression difference between ZS11 vs. the high-NPMI RNA pool and between the low-NPMI RNA pool vs. the high-NPMI RNA pool, respectively (Figure 2A). Ten genes were differentially expressed both in ZS11 vs. 73290 and in the low-NPMI vs. the high-NPMI RNA pool. In sum, we identified 4805 genes that were significantly differentially expressed among the four samples (Table S1).

**Figure 3:**
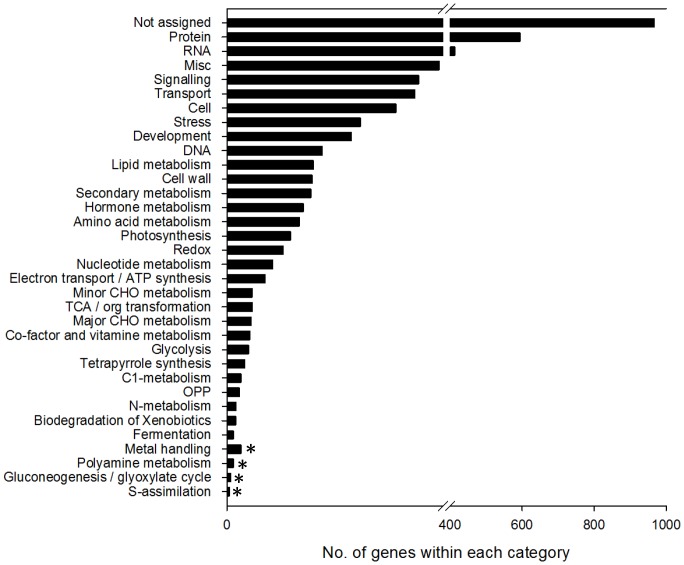
Functional categories of differentially expressed genes are significantly enriched using MapMan as a classification source. GO terms are sorted based on P value (p<0.05). * indicates the unenriched GO terms.

Furthermore, probe annotation analysis against TAIR10 indicated that 3981 of the 4805 DEGs matched at least one gene annotated in TAIR10, and 824 shared no significant homology with any Arabidopsis accession. The functional categories were determined with 100 bootstrap replicates against TAIR10 using MapMan as the classification source and produced a total of 31 enriched clusters (Figure 3). Among the enriched categories, we detected highly enriched clusters with strong confidence levels (p<0.05), such as carbohydrate synthesis and metabolism, development regulation, hormone responses, signal transduction, stress and redox, and transcription regulation (Table S1).

### Expression patterns of DEGs in the main inflorescence of *B. napus*

Next, we monitored the expression patterns of these 4805 DEGs in the MI using hierarchical cluster analysis. This revealed six major clusters (Figure 2B). Genes were subjected to functional category enrichment analysis in each cluster compared with their distribution among all genes in *Arabidopsis*. About 17.94% (862) of the DEGs occurred in cluster I and were up-regulated in the MI of ZS11 but down-regulated in that of the low-NPMI RNA pool, 73290, and high-NPMI RNA pool, while the mRNA abundance of these genes was similar in the MIs of 73290 and the high-NPMI RNA pool. Amongst these DEGs, several functional categories, such as protein and amino acid metabolism, nucleotide metabolism, cell organization, photosynthesis and mitochondrial electron transport, DNA duplication and repair, and transport, were significantly enriched.

In cluster II, which accounted for 22.33% (1073) of the DEGs, genes were upregulated in the MIs of ZS11and partially in the low-NPMI RNA pool relative to 73290 and the high-NPMI RNA pool, with the expression in the MI of the high-NPMI RNA pool being greater than that in 73290. These DEGs were mainly involved in protein and amino acid metabolism, transcription regulation, photosynthesis and mitochondria electron transport, carbohydrate metabolism, stress response, and transport, or had not been assigned to a functional group.

About 10.92% (525) of genes fell in cluster III and displayed significantly higher expression in line 73290 than in the high-NPMI RNA pool. In contrast, in cluster V, which accounts for 15.98% (768) of the DEGs, genes were expressed at higher levels in the high-NPMI RNA pool and lower levels in line 73290. Furthermore, the expression level of genes in clusters III and V was similar in the MI of ZS11 and the low-NPMI RNA pool. These genes were enriched in categories such as protein metabolism, transcription regulation, signaling, and transport.

Similarly, 449 (9.34%) of the DEGs were classified into cluster IV and these genes had lower expression in the high-NPMI RNA pool and line 73290 than in line ZS11 and the low-NPMI RNA pool, with expression being lowest in the high-NPMI pool. The genes in this category were mainly related to protein metabolism, transcription regulation, signal transduction, and lipid metabolism.

Cluster VI was the largest, consisting of 1128 genes and accounting for 23.48% of the DEGs. The expression of genes in this cluster was greatest in the low-NPMI RNA pool and weakest in ZS11, and was similar in 73290 and the high-NPMI lines. We found that 41.8% of the genes in this cluster had not been assigned functional roles or had no hits against TAIR10, and the remaining genes were involved in photosynthesis, cell wall formation, lipid metabolism, secondary metabolism, the stress response and other categories.

Taken together, these results suggest that most of the DEGs identified in the experiment are likely to be involved in MI and floral organ differentiation and development, and that most of the observed expression changes are a consequence of allele-specific gene expression among the two parents and their F_6_ RILs.

### Localization of DEGs in QTL-containing intervals

Although the genomic sequence of *B. napus* is currently incomplete, the genome of *B. rapa* has been sequenced [28] and that of *B. oleracea* is almost complete in OCRI-CAAS (Shengyi Liu et al., 2014). Since *B. rapa* (AA, 2n = 20) and *B. oleracea* (CC, 2n = 18) are the diploid ancestors of amphidiploid *B. napus* (AACC, 2n = 38), the 4805 DEGs identified in this study can be localized on chromosomes of *B. napus* by referencing the genome information of *B. rapa* and *B. oleracea* using BLASTn analysis at an *E*-value of <10^−5^. Consequently, we established that 4363 of the 4805 DEGs are specifically co-localized on *B. rapa* chromosomes and 4300 are on *B. oleracea* chromosomes. Furthermore, 4460 genes are distributed over all 19 linkage groups of *B. napus*, and the remaining 345 are currently homeless, and do not localize to any chromosomes of *B. napus*.

**Figure 4:**
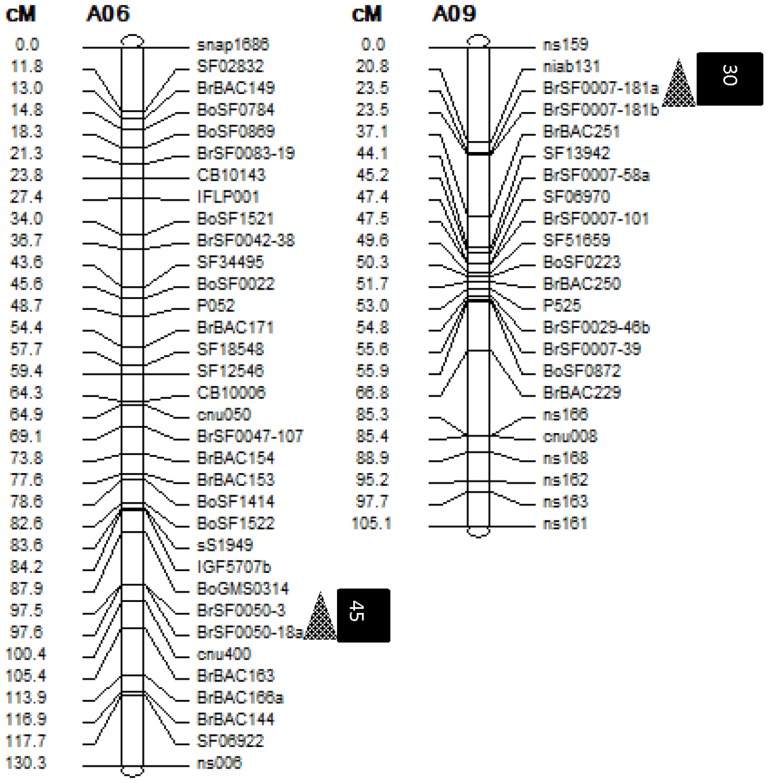
Mapped QTLs associated with the NPMI and DEGs that localized within the QTL intervals. Triangles indicate the mapped QTLs for NPMI and black squares with embedded digits denote the position and number of co-localized DEGs. A06 and A09 denote chromosomes A06 and A09 of *B. napus*, respectively.

A genetic map (whole length 1869.7 cM) containing 519 markers (average distance = 3.6 cM) was constructed using the F_2∶3_ population derived from the cross ZS11*73290. We detected two QTLs associated with the NPMI on chromosomes A06 in 2010 (PMI10) and A09 in 2011 (PMI11), with a 17.6% and 13.9% contribution rate, respectively (Table 1 and Figure 4). We found that 45 and 30 DEGs identified in the current study were co-localized to the QTL intervals *PMI10* and *PMI11* (Table S2), suggesting that these genes regulate pod differentiation and development on the MI in *B. napus*. Ten of these 75 mapped DEGs are novel, with no homology to sequences in *Arabidopsis*, and the remaining 65 have orthologs in *Arabidopsis* that are involved in several biological processes, including transcription regulation, cell division and organization, and carbohydrate metabolism (Table S2). Several DEGs that co-localized to QTL intervals and were up- or down-regulated in both ZS11 and the low-NPMI pool were selected for quick functional analysis by overexpression in *Arabidopsis*.

**Table 1:** Identified QTLs for pods on the main inflorescence and co-localized DEGs.

Date	QTLs	Chrom.	Interval	QTL position (cM)	LOD	Additive effect	Dominant effect	VE (%)	No. of co-localized genes
2010	PMI10	A06	97.6–105.4	99.7	9.8	−5.31	2.21	17.6	45
2011	PMI11	A09	20.8–23.5	23.5	4.6	−12.56	−9.03	13.9	30

### Functional analysis of *BnTPI* in *Arabidopsis thaliana*

**Figure 5:**
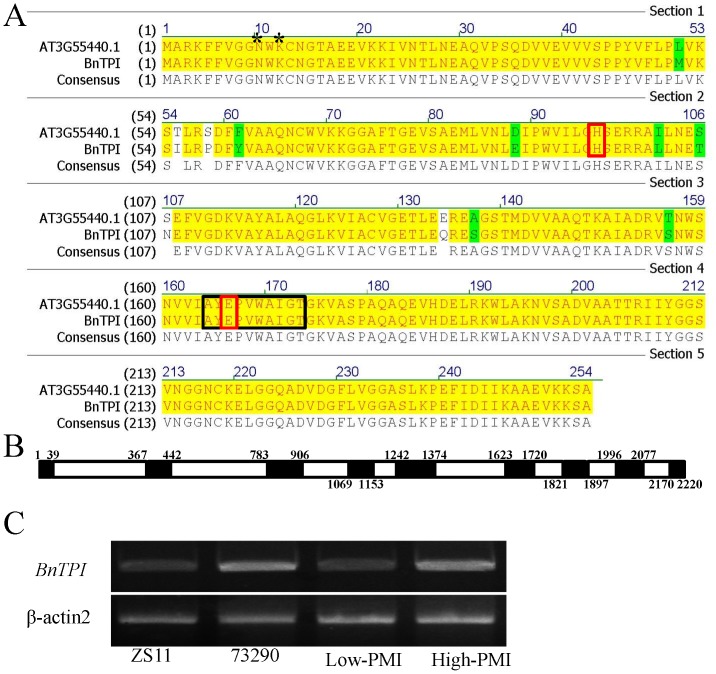
Alignment of the BnTPI and AtTPI amino acid sequences, gene structure prediction, and expression level validation using RT-PCR. (A) BnTPI displays a highly conserved domain (164–174 AA, black rectangular box), two binding sites (N10 and K12, black star), two active sites (H96 and E166, red box), an electrophilic site, and a proton acceptor point. Yellow and green shading indicate conserved and mutant amino acids between BnTPI and the *Arabidopsis* ortholog AT3G55440.1, respectively. (B) Schematic representation of *BnTPI*. Exons are shown as black boxes and introns as white boxes. Numbers above the boxes indicate the nucleobases (bp). (C) The expression level of *BnTPI* in the MI of the ZS11, 73290, and F_6_ RIL lines was determined by RT-PCR. *β-actin2* expression was used to normalize the transcript level in each sample.

Some of the DEGs transformed into *Arabidopsis* caused morphological variations in inflorescence traits. For instance, MI52894, a DEG that co-localized to a QTL interval on chromosome A6, was strongly up-regulated in the MI of both the high-NPMI pool and 73290. This gene had 96% amino acid identity with *Arabidopsis AtTPI* (AT3G55440), which encodes triosephosphate isomerase (TPI). We thus named this gene *BnTPI* (Figure 5A). The full-length ORF (765 bp) of *BnTPI* was isolated and sequenced in both ZS11 and 73290. *BnTPI* was composed of nine exons, and its CDSs from ZS11 and 73290 were identical (Figure 5B). The expression level of *BnTPI* in the MI was determined by RT-PCR (Figure 5C), and found to be up-regulated both in the high-NPMI pool and 73290 compared to the low-NPMI pool and ZS11.

**Figure 6:**
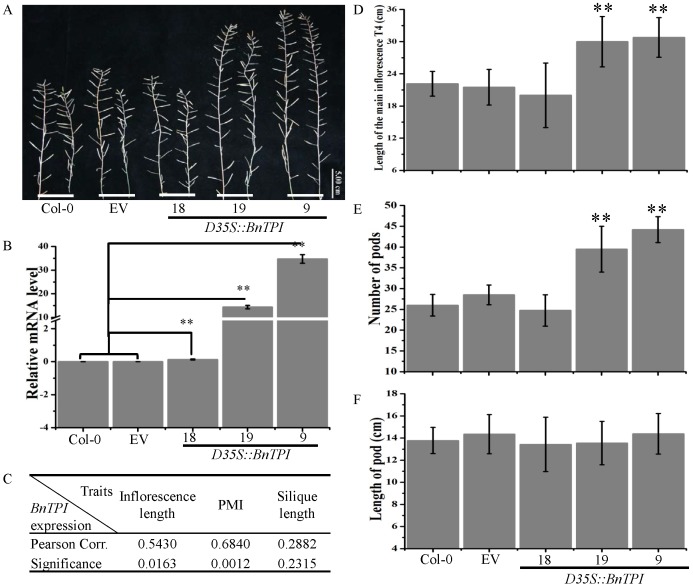
Features of the MI of wild type Col-0, EV, and *D35S::BnTPI* transgenic lines. (A) Morphological variations in the MIs and pods of the wild type Col-0, EV, and *D35S::BnTPI* transgenic lines. (B) Relative RNA abundance of *BnTPI* in the MI of wild type Col-0, EV, and *D35S::BnTPI* transgenic lines. The error bar is based on biological triplicates. (C) Pearson's correlation coefficient between *BnTPI* expression and inflorescence traits, including MI length, NPMI, and silique length; (D), (E), and (F) transgenic *D35S::BnTPI* and Col-0 and EV lines display significant differences in MI length, NPMI, and pod length, as determined by Student's *t*-test at the p<0.05 level. Phenotype variation of twenty plants per line was investigated.

Subsequently, we generated the *D35S::BnTPI* construct and transformed this construct and its corresponding empty vector (EV) into wild type *Arabidopsis* Col-0. Nineteen positive homozygous transgenic lines (T_4_) and their EV and wild type Col-0 controls were planted under identical conditions in a growth room and harvested for phenotype investigation. We found that 14 of the 19 *BnTPI* overexpression lines (T_4_) had 4 to 18 more pods on the MI than did the EV and Col-0 controls, and 10 transgenic lines produced MIs that were 0.5 to 9.3 cm longer than those on the EV control (Figures 6A, 6D, and 6E). Simultaneously, we examined the expression level of *BnTPI* in 9-day-old seedlings of the positive transgenic, EV, and Col-0 lines using real-time PCR with a pair of gtpi primers (Table S3). We found that the transcript abundance of *BnTPI* (relative to the expression level of *ACTIN2* (AT3G18780)) was significantly higher in the positive transgenic line than in the Col-0 and EV plants (Figure 6B). Moreover, the expression level of *BnTPI* was positively correlated with the length of the MI and the NPMI (Figures 6C, 6D and 6E), while no obvious correlation occurred between *BnTPI* expression and pod length on the MI (Figures 6C and 6F).

**Figure 7:**
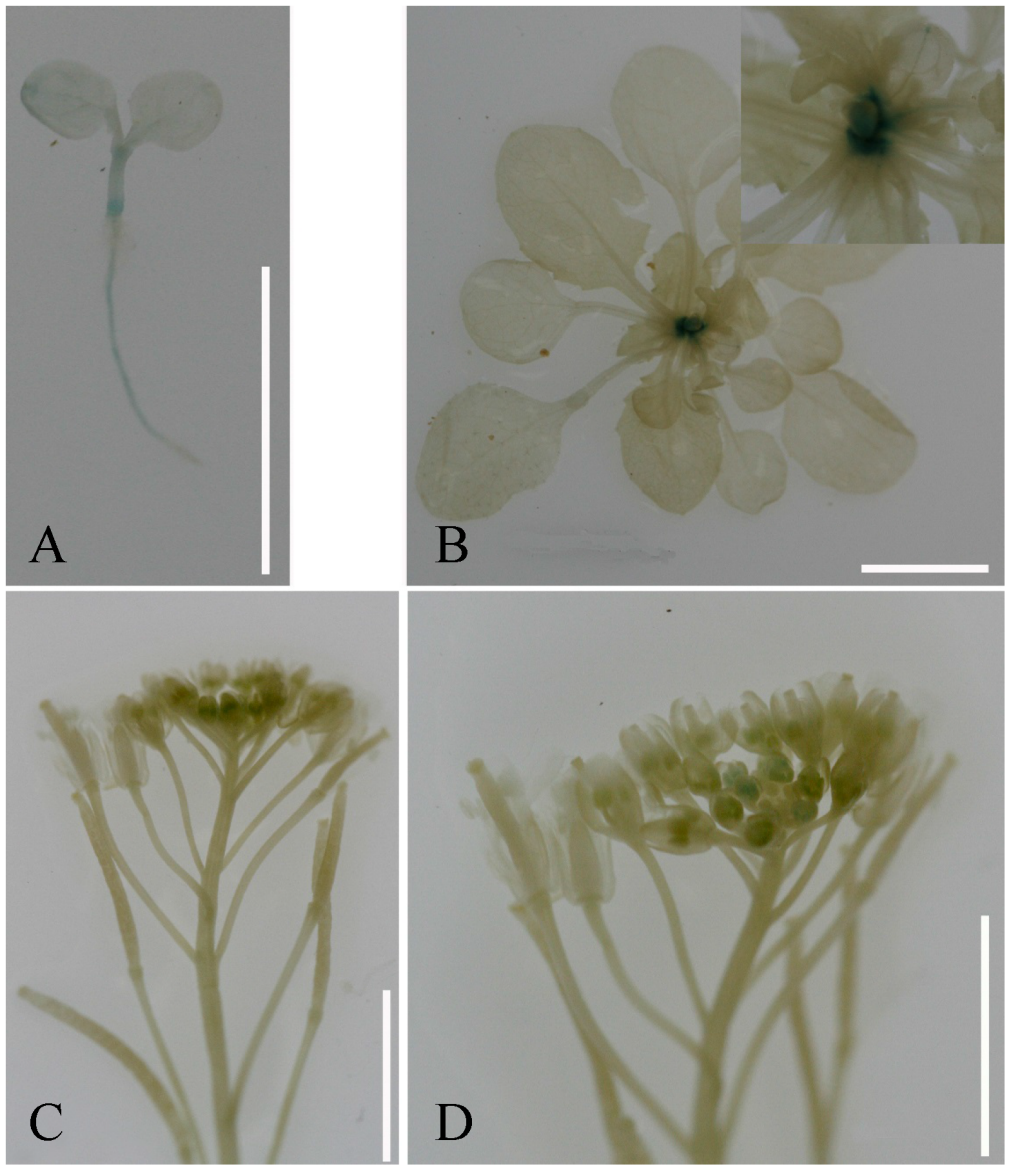
Histochemical staining of GUS activity in *pBnTPI::GUS* transgenic *Arabidopsis* lines. (A) In nine-day-old seedlings, GUS activity was detectable at the shoot apex meristem (SAM), node-joint, and roots. (B) GUS activity in the rosette axils of 24-day-old plants. The insets on the top right corner indicate the megascopic rosette. (D) Weak GUS activity in the SAM and floral buds at flowering, and (E) a zoomed-in view of the stained floral buds.

Furthermore, we cloned and sequenced the *BnTPI* promoter from ZS11 and 73290, but did not detect any differences between the two. We monitored the tissue-specific activity of the *BnTPI* promoter by generating a translational fusion construct *pBnTPI::GUS* and transforming it into *Arabidopsis* Col-0 plants. Twelve independent *pBnTPI::GUS-*positive transgenic lines were obtained, and the GUS activity was found to vary throughout development in a tissue-specific manner. In nine-day-old transgenic seedlings, GUS staining was clearly visible in the shoot apex meristem (SAM), node-joint, and roots (Figure 7A), whereas in 24-day-old transgenic plants, it was mainly confined to the axil and the SAM (Figures 7B and 7C), and at flowering, weak GUS staining was detectable in the SAM and floral buds (Figures 7D and 7E).

## Discussion

The *B. napus* inflorescence is a raceme in which the apical meristem is able to grow indefinitely, generating a continuous main axis that laterally produces floral meristems. The MI plays a determinant role in the reproductive success of *B. napus* [29]. In the current study, in an attempt to identify the genes underlying pod differentiation and development on the MI in *B. napus*, we analyzed the gene expression profiles in the MIs of two F_6_ RIL populations with great differences in pod number and their corresponding elite parental cultivars using an oligonucleotide array. The analysis was performed when the first flower on the MI was in the pistil-stamen primordial differentiation state, as the NPMI is directly correlated with the number of differentiated floral primordia, and it is easy to synchronize the development of different plants. The expression of about 5% of transcripts differed significantly between the low-NPMI and high-NPMI samples, and these transcripts were involved in multiple pathways. This analysis provided a considerable amount of information that can be used to assess patterns of gene expression that may be relevant for determining pod differentiation and development on the MI of *B. napus*.

A remarkable outcome of this study was the finding that a considerable proportion of the genes in the four RNA samples exhibited significant differences in expression level. These differences were displayed in several forms: the genes may be highly expressed in one of the parents, but expressed at low levels in the other parent and F_6_ lines, or highly expressed in both low-NPMI samples, but expressed at low levels in the high-NPMI samples and vice versa, or highly expressed in both extreme F_6_ line pools, but expressed at low levels in either or both parents. The expression level of some genes was altered among the four RNA samples, but not at a significant level (i.e., <1.5-fold), and we would like to examine the potential involvement of these genes in pod differentiation and development as well. The growth and development of organisms is widely believed to be spatially and temporally regulated at the transcriptional level. This study shows that a gene may be strongly expressed in the MI of one genotype, but very weakly expressed in another, resulting in large differences in the level of gene expression among the genotypes. These differences in gene expression provide insight into the mechanisms that regulate pod differentiation and development.

Recent studies showed that quantitative variations in gene expression levels were controlled genetically and were regulated by both *cis*- and *trans*-acting loci, with *trans*-acting elements playing the dominant roles [30], [31]. We thus selected DEGs as candidate regulators of NPMI and rapidly validated the functions of ten gene through overexpression analysis in *Arabidopsis*. Our analysis of DEGs between the two parental lines revealed that sequence differences in the CDS, promoter, or both regions are common, and that the sequence differences of several DEGs result in obvious phenotypic variation in the NPMI and other floral organs in *Arabidopsis* (data no shown), suggesting that these phenotypic variations are attributable to *cis*-acting regulation or to variations in the CDSs of genes between the two genotypes. These findings are consistent with previous studies in other organisms [32]–[35]. In addition, we identified some DEGs, such as *BnTPI*, which exhibited no sequence differences in either the CDS or promoter regions, but displayed obvious phenotypic variation in the NPMI and other floral organs, indicating that *trans*-acting elements play important roles during pod differentiation in *B. napus*. We established that 5% of *B. napus* genes were differentially expressed among the four RNA samples. We plan to determine the cause of the observed expression differences by characterizing more of the DEGs, as this would enhance our fundamental understanding of mechanisms underlying differences in the NPMI in *B. napus*.

It was suggested that gene expression data together with QTL analysis may provide an avenue for identifying candidate genes for traits of interest [36]. In this analysis, we therefore also attempted to establish a link between the DEGs and potential QTLs related to the NPMI. We identified two QTLs associated with the NPMI using an F_2∶3_ population derived from a cross between the parents used in this study, and 4460 of the 4805 DEGs were mapped to 19 chromosomes of *B. napus* referencing genome sequences of both *B. rapa* and *B. oleracea*. Among the mapped DEGs, 45 and 30 were localized to the two QTLs for NPMI spanning 7.8 cM and 2.7 cM, respectively (Table 1). As the ORF regions of *Arabidopsis thaliana* and *B. napus* exhibit an estimated 85% sequence identity [37], it would be helpful to identify the homologs of *Arabidopsis* genes known to control floral and inflorescence differentiation in *B. napus*. The functional roles of a representative DEG were further investigated, with overexpression in *Arabidopsis* resulting in an increased NPMI and longer inflorescences. We identified *BnTPI* as a factor in pod determination on the MI and, based on the function of the *Arabidopsis* homolog, suggest that energy harvesting, reservation, and conversion contribute to inflorescence differentiation, elongation, and floral bud procreation during flowering in *B. napus*. Thus, this study identified several likely candidate genes that affect the NPMI, and these genes warrant further studies.

It should be pointed out that the current study only established the landscape of gene expression in the *B. napus* MI using an oligonucleotide array that was developed based on non-overlapping *Brassica* EST contigs and singletons that ideally represent unique gene models. Along with the full-scale genome sequencing of *B. napus* and *B. oleracea* and advances in micro-technologies, it would be helpful to decipher all potential genes that affect the NPMI in the genomes of *B. rapa*, *B. oleracea*, and *B. napus* and to place the entire set of *Brassicaceae* genes on a single chip. This comprehensive chip would provide an effective approach for establishing extensive expression profiles throughout the life cycle of *B. napus*. All genes that affect the NPMI and other inflorescence traits would certainly be identified using such a chip, and the roles of these genes could be gradually determined using functional assays.

## Conclusions

We report here the first comprehensive survey of gene expression profiles of the *B. napus* MI. The datasets created in this study exhibited variation at the gene expression level that is intricate, but coordinated. Several expression patterns and prominent processes and pathways in the MI were identified as increasing pod quantity and the duration of inflorescence differentiation in *B. napus*. The comparison of DEGs with NPMI QTLs suggested a number of likely candidates that warrant further studies. Our initial analysis of the functions of *BnTPI* and other interesting genes in *Arabidopsis* provides a starting point for further investigations into the regulation of pod primordia initiation, maintenance of SAM duration, and even control of seed yield in *B. napus*. These findings contribute to our understanding of the mechanisms underlying pod and inflorescence differentiation and development, and pave the way for developing molecular breeding strategies for improved yield *B. napus* and other crops.

## Supporting Information

Table S1 **A list of 4805 significantly differentially expressed genes with their signal ratios and best BLASTx annotation.**
(XLSX)

Table S2 **A list of co-localized DEGs in the QTL intervals for NPMI.**
(XLSX)

Table S3 **A list of primers of differentially expressed genes used for quick functional analysis in** ***Arabidopsis*** **.**
(XLSX)
